# Supplementary material for: Prognostic Effects of Vasomotor Reactivity during Targeted Temperature Management in Post-Cardiac Arrest Patients: A Retrospective Observational Study
Source: J Clin Med. 2021 Jul 30;10(15):3386. doi: 10.3390/jcm10153386 (PMC8348065; doi:10.3390/jcm10153386)
Supplement: Supplementary file 1 [file jcm-10-03386-s001.zip › jcm-1268971-supplementary.pdf]

Table S1. Comparision with normal control (age/sex matched).

	Normal control (n = 37)	Good outcome (n = 39)	Poor outcome (n = 76)	p from Kruskal-Wallis	p Normal vs. good	p Normal vs. poor
Age, yearr	56 (44-64)	56 (40.5–62)	64 (52–75.5)	0.003	0.888	0.030
Sex, male	23 (62.2)	32 (82.1)	49 (64.5)	0.102		
Baseline mean flow velocity, cm/s	56.1 ± 13.2	51.9 ± 21.1	69.4 ± 28.1	0.002	0.064	0.042
Baseline mean pulsatility index	0.77 ± 0.13	0.80 ± 0.25	0.85 ± 0.33	0.911		
Mean VMR, %	29.0 ± 7.6	26.0 ± 15.8	12.8 ± 13.2	<0.001	0.036	<0.001
Mean VMR per change of PaCO2, %/mmHg	5.54 ± 2.82	7.45 ± 10.10	2.38 ± 3.29	<0.001	<0.001	<0.001

This comparison was conducted on patient with transcranial Doppler test.VMR = Vasomotor reactivity.

Table S2. Prognostic performance to predict good CPC at 1month.

Variables	TP	TN	FP	FN	Sensitivity	Specificity	PPV	NPV
Presence of pupillary light reflex	38	65	52	3	92.7	55.6	42.2	95.6
GCS motor score ≥ 2	16	103	14	25	39.0	88.0	53.3	80.5
GWR ≥ 1.17	34	65	52	7	82.9	55.6	39.5	90.3
ASPECTS-b ≥ 7	36	69	48	5	87.8	59.0	42.9	93.2
Initial S100 < 2.37 (1 missing)	29	82	35	11	72.5	70.1	45.3	88.2
VMR ≥ 30.3 *	32	58	18	7	82.1	76.3	64.0	89.2

TP = true positive, TN = true negative, FP = false positive, FN = false negative, PPV = positive predictive value, NPV = negative predictive value, GCS = Glasgow Coma Scale, ASPECTS-b = bilateral Alberta Stroke program early CT score, VMR = vasomotor reactivity, * TCD analysis: 115 patients (43 excluded; both poor temporal window, reverberating flow).
